# Altered Nitrogen Balance and Decreased Urea Excretion in Male Rats Fed Cafeteria Diet Are Related to Arginine Availability

**DOI:** 10.1155/2014/959420

**Published:** 2014-02-24

**Authors:** David Sabater, Silvia Agnelli, Sofía Arriarán, José-Antonio Fernández-López, María del Mar Romero, Marià Alemany, Xavier Remesar

**Affiliations:** ^1^Department of Nutrition and Food Science, Faculty of Biology, University of Barcelona, 08028 Barcelona, Spain; ^2^Institute of Biomedicine, University of Barcelona, 08028 Barcelona, Spain; ^3^CIBER Obesity and Nutrition, Institute of Health Carlos III, 28029 Madrid, Spain

## Abstract

Hyperlipidic diets limit glucose oxidation and favor amino acid preservation, hampering the elimination of excess dietary nitrogen and the catabolic utilization of amino acids. We analyzed whether reduced urea excretion was a consequence of higher NO_*x*_; (nitrite, nitrate, and other derivatives) availability caused by increased nitric oxide production in metabolic syndrome. Rats fed a cafeteria diet for 30 days had a higher intake and accumulation of amino acid nitrogen and lower urea excretion. There were no differences in plasma nitrate or nitrite. NO_*x*_ and creatinine excretion accounted for only a small part of total nitrogen excretion. Rats fed a cafeteria diet had higher plasma levels of glutamine, serine, threonine, glycine, and ornithine when compared with controls, whereas arginine was lower. Liver carbamoyl-phosphate synthetase I activity was higher in cafeteria diet-fed rats, but arginase I was lower. The high carbamoyl-phosphate synthetase activity and ornithine levels suggest activation of the urea cycle in cafeteria diet-fed rats, but low arginine levels point to a block in the urea cycle between ornithine and arginine, thereby preventing the elimination of excess nitrogen as urea. The ultimate consequence of this paradoxical block in the urea cycle seems to be the limitation of arginine production and/or availability.

## 1. Introduction

Metabolic syndrome is a pathological condition, which develops from localized inflammation and is characterized by the combination of a number of closely related diseases (insulin resistance, obesity, hyperlipidemia, hypertension, etc.) [1]. Administration of “cafeteria” diets [2] to rats has been used as an animal model for the study of late-onset hyperphagic obesity and metabolic syndrome. This model has the advantage of being comparable to some human obesity states induced by the excessive intake of energy-dense food [3, 4]. The effects of the cafeteria diet are more marked in males [5, 6]; this may be because they have less anti-inflammatory [7] estrogen protection than females. Low estrogen levels render males more prone to be affected by glucocorticoids [8, 9], which in turn decrease the anabolic effects of androgens [10, 11].

In rodents, prolonged exposure to a cafeteria diet results in higher energy intake (mainly lipids) [5] and increased body fat (obesity) but also affects lean body mass, favoring growth and protein deposition [12, 13]. Although the hedonic component of the cafeteria diet initially elicits an increase in food consumption [14], once obesity is well established, hyperphagia decreases to almost normal levels of food intake. However, the large mass of accumulated fat remains, and the metabolic consequences of excess energy intake—such as insulin resistance and hyperlipidemia—persist [6, 15].

The study of nitrogen handling under hypercaloric diet conditions has predominantly been limited to measuring plasma amino acid levels [16–18], while less attention has centered on pathways [19, 20] and nitrogen balances [21]. To date, most research on dietary amino acid metabolism has been directed toward the analysis of metabolic adaptation to diets deficient in both energy and amino nitrogen [22–25] or has focused on specific regulatory pathways. Cafeteria diets are suitable for the study of the effects of high-energy diets, because total protein intake is practically unaffected by the excess dietary energy (mainly lipids) ingested [5].

Dietary or body-protein amino acid nitrogen is spared when other energy sources (such as fat or glucose) abound. Thus, high-energy diet, such as the cafeteria diet, apparently decreases overall amino acid catabolism, inducing a marked decrease in the production of urea [26]. The relative surplus of 2-amino nitrogen can maintain protein turnover and growth [12], but the excess nitrogen must be excreted in some way. The limited operation of the urea cycle suggests that there may be more amino nitrogen available for the operation of the nitric oxide (NO^∙^) shunt. Obese humans have been found to excrete more nitrate than their lean counterparts, and loss of NO^∙^ in expired air is proportional to body mass index (BMI) [27]. In terms of nitrogen balance, it has been found that cafeteria diet-fed rats show a higher “nitrogen gap,” that is, the difference between nitrogen intake and the sum of its accumulation and excretion in the urine and feces [21] than control diet-fed animals.

The objective of the present study was to determine whether cafeteria diet-fed rats show changes in the excretion of nitrate/nitrite in comparison with age-matched animals fed standard rat chow, investigating whether an increase in the excretion of NO_*x*_ compensates for the decrease in urea excretion.

## 2. Materials and Methods

### 2.1. Animals and Experimental Setup

All animal handling procedures and the experimental setup were carried out in accordance with the animal handling guidelines of the European, Spanish, and Catalan Authorities. The Committee on Animal Experimentation of the University of Barcelona authorized the specific procedures used. This limited keeping the animals in metabolic cages to a maximum of 24 h to prevent unacceptable levels of stress.

Nine-week-old male Wistar rats (*N* = 12) (Harlan Laboratories Models, Sant Feliu de Codines, Spain) were used. The animals were randomly divided into two groups and were fed *ad libitum*, for 30 days on either normal rat chow (Harlan 2014) (*N* = 6) or a simplified cafeteria diet (*N* = 6) [21]. Both groups were housed in solid-bottomed cages with three animals per cage, had free access to water, and were kept in a controlled environment (lights on from 08:00 to 20:00, with a temperature of 21.5–22.5°C; 50–60% humidity). Body weight and food consumption were recorded daily. Calculation of food ingested was performed as previously described by counting the difference between food offered and left, including the recovery of small pieces of food, and compensating for drying [5]. The nitrogen content of the rat chow and the different components used in the cafeteria diet were measured with a semiautomatic Kjeldahl procedure using a Pro-Nitro S semiautomatic system (JP Selecta, Abrera, Spain).

On day 0 (i.e., the day before the experiment began) and day 26, the rats were kept for 24 h in metabolic cages (Tecniplast Gazzada, Buguggiate, Italy), recovering the urine and feces. In the metabolic cages, all rats were fed only standard rat chow and tap water, and their food consumption was measured. Samples of excreta were frozen for later analyses. Urine NO_*x*_ was estimated immediately to minimize further oxidation and NO^∙^ losses, using a nitric oxide analysis system (ISM-146NOXM system) (Lazar, Los Angeles, CA, USA).

On day 30, rats were anesthetized with isoflurane and then killed by exsanguination through the exposed aorta. Blood plasma and tissue samples were obtained and frozen. Liver samples were rapidly frozen in liquid nitrogen and maintained at −80°C until processed for enzyme analyses. For tissues distributed widely throughout the body (i.e., subcutaneous adipose tissue), all the tissue was carefully dissected and weighed. Hind leg muscle samples were cut from the hind leg, obtaining part of the *quadratus femoris, biceps femoris*, and *semitendinosus *muscle and a smaller proportion of others.

### 2.2. Diet Composition

In the standard diet (Harlan 2014), 19.9% of energy was derived from protein, 13.8% from lipids, and 65.8% from carbohydrates (10% from sugars).

The cafeteria diet included biscuits spread with liver pâté, bacon, standard chow pellets, water, and milk supplemented with 300 g/L sucrose plus 10 g/L of a mineral and vitamin supplement (Meritene, Nestlé, Esplugues, Spain). All of these compounds were provided fresh daily. From the analysis of the diet components and the ingested items, we calculated that a mean of 33% of energy was derived from lipids, 16% of energy was derived from protein, and 51% of energy was derived from carbohydrates (20% from sugars).

### 2.3. Body and Metabolite Analyses

Total body muscle mass was estimated, as previously described [28], using the remaining carcass. The method was based on the solubilization of muscle actin and myosin with 1 M LiCl and subsequent precipitation of mainly myosin with distilled water, followed by its estimation with a standard procedure.

Stool nitrogen was measured using the semiautomatic Kjeldahl procedure described above. Nitrogen content and nitrogen accrual were calculated by applying the body composition factors obtained from previous studies [3, 21] to our experimental animals. These data are included as reference values only for comparison. Urine urea was measured with a urease-based test, and creatinine was measured with the Jaffé reaction using commercial kits (BioSystems, Barcelona, Spain).

Plasma was used for the analysis of glucose, total cholesterol, triacylglycerol and urea (using kits from BioSystems, Barcelona, Spain), nonesterified fatty acids (NEFA kit; Wako, Richmond VA, USA), and L-lactate (Spinreact kit, Barcelona, Spain). Nitrite and nitrate were measured with the ArrowStraight system. Plasma samples were used for amino acid quantification after deproteinization with trifluoroacetic acid; they were measured with ninhydrin, in a Biochrom 30 autoanalyzer (Biochrom, Cambridge, UK), using L-norleucine as internal standard, at the Scientific-Technical Services of the University of Barcelona.

### 2.4. Enzyme Assays

Frozen liver samples were homogenized in chilled 50 mM Krebs-Ringer phosphate buffer, pH 7.4, containing 0.1% Triton X-100, 2.5 mM mercaptoethanol, 0.1% dextran, 5 mM Na_2_-EDTA, and 0.5% bovine serum albumin using a mechanical tissue disruptor (IKA, Staufen, Germany). Arginase I (EC 3.5.3.1) activity was estimated as described elsewhere [29]. The method was based on the colorimetric estimation of urea (Berthelot reaction) released by the action of arginase on arginine. Homogenate protein content was measured with a standard colorimetric method [30] against blanks of the homogenization medium.

Other liver samples were homogenized in 50 mM triethanolamine-HCl buffer, pH 8.0 containing 1 mM dithiothreitol, and 10 mM magnesium acetate. Carbamoyl-P synthetase I (EC 6.3.4.16) activity was measured immediately after homogenization, as previously described [31], by measuring the incorporation of labeled bicarbonate (50 kBq/mmol) into carbamoyl-P in a medium containing 5 mM ATP, 5 mM N-acetyl-glutamate, and 0.05% bovine serum albumin. Enzyme activities were expressed in katals both in reference to the weight of fresh tissue and its protein content.

### 2.5. Statistical Analysis

Statistical analysis was carried out using one-way ANOVA, with the *post hoc* Bonferroni test, and/or the unpaired Student's *t*-test, using the Statgraphics Centurion XVI software package (StatPoint Technologies, Warrenton, VA, USA).

## 3. Results

Table 1 shows the body weights and nitrogen balance values for control and cafeteria diet-fed rats at the beginning and end of the study (day 27 for nitrogen data). As expected, the increase in body weight was greater in cafeteria diet-fed rats than in controls. The overall energy and nitrogen intake were also higher in cafeteria diet-fed than in control rats.

In the period that the rats were kept in metabolic cages, significant differences in nitrogen intake were observed between the groups, but not in urine or stool nitrogen excretion. The proportion of urea excreted with respect to the total daily nitrogen budget was lower in cafeteria diet-fed rats than in controls on day 27 (65% versus 75% of nitrogen intake, 82% versus 94% of urea excreted, resp.). This difference was not compensated for by creatinine and NO_*x*_ excretion, which was low in comparison to urea, and showed slight changes over time or with dietary treatment. Thus, although the data on nitrogen balance was measured on different days, the estimated “nitrogen gap” showed a wider margin for cafeteria diet-fed rats than for controls.

The effects of diet on organ weight on day 30 are presented in Table 2. The only significant differences between the two groups in organ weights were for muscle, stomach, heart, and adipose tissues. The other organs showed remarkably similar weights.

The plasma nitrate and nitrite concentrations are presented in Table 3. There were no significant differences between control and cafeteria-fed rats for nitrate, nitrite, or their sum. In both groups, however, nitrate was the predominant component (>90%).

Table 4 shows the plasma amino acid concentrations of the control and cafeteria diet-fed groups on day 30. The similarity between the groups was remarkable, with only a few amino acids showing significant differences. Cafeteria diet-fed rats had higher levels of glutamine, threonine, serine, glycine, and ornithine than controls, while the latter showed higher levels of arginine with respect to the cafeteria diet-fed animals. When analyzing the sums of concentrations of groups of related amino acids, no changes were observed for the combined concentrations of glutamate + glutamine, aspartate + asparagine, branched-chain amino acids (leucine + isoleucine + valine), or urea cycle intermediaries (ornithine + citrulline + arginine).

The plasma concentrations of glucose, triacylglycerols, total cholesterol, and urea for both diet groups (Table 5) were similar, and all were within the normal range. These concentrations were similar to data previously published by our group, with no differences between the groups, except for higher glucose and lower urea values in cafeteria diet-fed rats.

**Figure 1:**
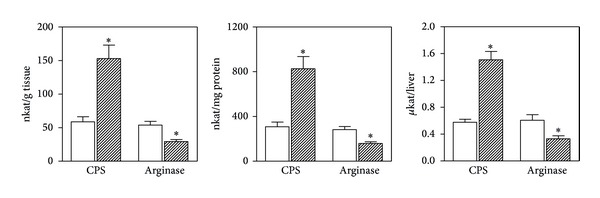
Carbamoyl-P synthetase I and arginase activities in the liver of rats fed a control or cafeteria diet for 30 days. CPS = carbamoyl-P synthetase I. Control: white columns; cafeteria diet-fed: dashed columns. The values are the mean ± SEM for 6 different animals. Comparisons between groups were established with Student's *t*-test: *P < 0.05.

Figure 1 presents the measured activities of two key urea synthesis enzymes in the livers of the control and cafeteria diet-fed groups. The activity of carbamoyl-P synthetase I was higher than that of arginase I in the cafeteria diet-fed group; these rats had threefold higher activity rates for carbamoyl-P synthetase I than controls. The results for arginase were the reverse, since the control group had almost twice the activity per unit of tissue weight than the cafeteria diet-fed group, and this result was similar for protein and total tissue.

## 4. Discussion

The cafeteria diet is essentially hyperlipidic, with identical mean protein and carbohydrate intakes to those of control rats fed a standard diet [5]. As expected, a one-month exposure to a cafeteria diet resulted in overfeeding and increased body weight, leading to a greater increase in the size of adipose tissue deposits and higher muscle mass than in controls. These results are in agreement with previous studies showing that, as with the hyperlipidic diets, a cafeteria diet increases not only fat deposition and growth, but also protein accrual [12, 21] and energy expenditure [32]. A lower excretion of urea, irrespective of the maintained (or increased) amino acid intake, was also observed, again in agreement with previous studies [21, 26].

It has been postulated that a high-energy diet coupled with normal or increased protein intake may hamper 2-amino nitrogen elimination in rats, humans, and other mammals [33]. This problem is largely a consequence of the abundance of energy, mainly in the form of lipids, which is used preferentially by muscle and other peripheral tissues over glucose because of insulin resistance [34]. However, amino acid oxidation is spared due to the availability of energy, that is, in the form of glucose [35]; thus, is to be expected that catabolism of dietary amino acids and, therefore, the production of ammonium from 2-amino nitrogen should also decrease. Consequently, during this buildup, the mechanisms of amino nitrogen waste prevention surprisingly create a surplus of available amino acids. The excess of 2-amino nitrogen may be limited, in part, by increased growth (e.g., increased muscle mass) and, to a lesser extent, by increased protein turnover. However, the problem remains that not enough ammonium can be produced from the amino acid pool to maintain the glutamine (or free ammonium) necessary for the splanchnic organs (i.e., the intestines, liver, and kidney) to eliminate the excess of nitrogen as urea [36–38].

The observed plasma levels of amino acids seem to confirm these trends. In cafeteria diet-fed rats, glutamine but not glutamate + glutamine levels were higher than in control rats. High levels of glutamine suggest its decreased splanchnic utilization to provide ammonium for the synthesis of carbamoyl-P. The high circulating levels of ornithine again suggest a diminished production of carbamoyl-P, perhaps because of scarcity of ammonium donors in the liver. The lower arginine levels in the cafeteria diet-fed group versus controls suggest that synthesis of arginine may be insufficient to compensate for the release of urea through arginase activity or other uses. Arginase I in liver, which is the main site for this enzyme to complete the urea cycle [39], showed lower activity in the cafeteria diet-fed group. This may help maintain circulating arginine, although at levels lower than in control-fed animals.

The higher carbamoyl-P synthetase I activity found in cafeteria diet-fed rats agrees with the clear surplus of 2-amino nitrogen available for excretion, since higher liver ammonium availability increases the activity of this enzyme [40]. The decrease in urea excretion agrees with lower arginase activity, but not with the increased activity of carbamoyl-P synthetase I, which depends on ammonium as its substrate [40]. Thus, the block in urea cycle function (and consequently in “normal” 2-amino nitrogen disposal) should lie between these enzymes in the urea cycle, that is, in the conversion of ornithine to citrulline or the latter to arginine (i.e., argininosuccinate synthetase and argininosuccinate lyase). In addition, the higher ornithine levels in the plasma of cafeteria diet-fed rats suggest that the N-acetyl-glutamate pathway for the exogenous synthesis of ornithine was not sufficiently activated to compensate for the arginine deficit.

Because the circulating levels of citrulline and aspartate were unchanged (or increased) in cafeteria diet-fed versus control rats, it can be assumed that there is probably a key regulatory path, for overall nitrogen disposal, either at the synthesis or breakup of argininosuccinate, which would help explain the lower production of urea. Based on kinetic studies, argininosuccinate synthesis was initially postulated as a key urea cycle control node [41], although normal urea cycle operation is assumed to rely more on other parameters such as pH, ammonium availability, and N-acetyl-glutamate levels [42]. However, the indirect data presented here suggest that argininosuccinate synthesis/breakup may be a significant control point *in vivo* under relatively high nitrogen (and energy) availability.

The involvement of ammonium availability in this context is enhanced by the relatively higher concentrations of threonine, serine, glycine, and glutamine in cafeteria diet-fed rats. These amino acids yield ammonium in their catabolism [43] via threonine/serine dehydratase, glutaminase, or the glycine cleavage system. Serine may also be converted to glycine, which leads to the same fate. These results show an unexpected picture, since, in cafeteria diet-fed rats, there is an excess of 2-amino nitrogen and the higher levels observed correspond to ammoniagenic amino acids. According to the normal catabolic pathways for nitrogen excretion, this excess should activate the production of ammonium, its transport as glutamine, release again as ammonium, and formation of carbamoyl-P, followed by its integration (with more aspartate-derived amino nitrogen) into the guanido group of arginine for its eventual release as urea. However, the amino acids that can yield ammonium directly, in an initial nontransaminative catabolic step, were somehow preserved in cafeteria diet-fed rats. These amino acids were not used in large quantities as was to be expected in a situation in which, theoretically, the lack of 2-amino nitrogen conversion to ammonium could hinder normal nitrogen excretion through the urea cycle. The contrast between preservation of the ammonium donors and high carbamoyl-P synthetase I activity in the cafeteria diet-fed group suggests that the problem does not lie in the availability of ammonium. Higher levels of the main amino acid ammonium donors suggest instead a constraint on their utilization because elimination via the urea cycle is blocked as indicated above.

The faulty operation of the urea cycle, then, suggests that the main control mechanism sought is not centered on the availability of ammonium-yielding substrates as is usually postulated for normal and starvation conditions [42]. The increased activity of carbamoyl-P synthetase and the low activity of arginase in cafeteria diet-fed rats indicate that the control mechanism lies in the actual synthesis of arginine, which is also essential for the operation of the NO^∙^ shunt. Notwithstanding, the NO^∙^ shunt does not seem to be significantly altered by the cafeteria diet, as shown by unchanged plasma and urinary NO_*x*_ in spite of lower circulating arginine. One possible explanation is that the blockage of arginine production results from the need to prevent an increase in the production of NO^∙^ under cafeteria diet conditions, in which blood flow—in part dependent on NO^∙^ synthesis—to a number of tissues is markedly altered [44].

The question remains of how the excess nitrogen provided by cafeteria diets is eliminated. The widening of the nitrogen gap under high-energy feeding suggests that nitrogen gas [45] may be involved, since the amount of creatinine, uric acid, and so forth, excreted is only a small fraction of urea nitrogen [46, 47]. The synthesis of NO^∙^ results in the excretion, mainly via saliva [48], of nitrite and nitrate. In addition, there is a small direct loss of NO^∙^ in the breath [49]. However, the low levels of NO_*x*_ measured in the urine and their marked metabolic effects [50] suggest that NO_*x*_ as NO^∙^ derivatives, could account for at most only a very small part of the “missing” nitrogen. The lack of changes elicited by diet in circulating levels of nitrate and nitrite reinforced this assumption; that is, nitrate excretion is not a significant alternative as a nitrogen-disposal pathway to lower urea synthesis.

The one-month period of exposure to the cafeteria diet proved that this type of diet caused difficulties in the normal mechanisms of amino nitrogen disposal, exemplified by a lower urea production. These problems were not directly related to the potential availability of ammonium as the prime substrate for initiating the urea cycle but instead were probably related to the availability of arginine. No changes were observed in the levels or excretion of NO_*x*_, which were small, but the “nitrogen gap” [21] became significant under cafeteria diet feeding. It is now clear that the decrease in urea excretion is not compensated for by higher NO_*x*_ production and elimination. The main pathway for disposal of the excess amino nitrogen generated by energy rich diets remains unsolved, with the additional conundrum of why the urea cycle appears to be disrupted for the only apparent reason of limiting the availability of arginine.

## 5. Conclusions

The decrease in urea excretion is not compensated for by higher NO_*x*_ production and elimination. The defective operation of the urea cycle in rats fed a cafeteria diet seems to be caused by a block in the urea cycle between ornithine and arginine.

## Figures and Tables

**Table 1:** Energy and nitrogen balances of male rats fed a cafeteria diet compared with controls fed a standard rat chow diet.

	Units	Control: initial (*n* = 12)	Control: final (*n* = 6)	Cafeteria diet-fed: final (*n* = 6)	*P*	Control: (days 1–30)	Cafeteria diet-fed: (days 1–30)
Rat weight	g	291 ± 2^A^	373 ± 17^B^	449 ± 8^C^	<0.001		
Rat weight change	g/30 day					83 ± 7	158 ± 7*
Energy intake	kJ/d	326 ± 6^A^	365 ± 6^A^	596 ± 11^B^	<0.001	350 ± 9	680 ± 6*
Body nitrogen content	g	10.4 ± 0.7^A^	13.0 ± 0.8^AB^	15.0 ± 1.2^B^	0.004		
Body nitrogen accrual	mg/day					87 ± 8	157 ± 11*
Nitrogen intake	mg/day	478 ± 17^A^	481 ± 14^A^	652 ± 25^B^	<0.001	475 ± 11	813 ± 13*
Stool nitrogen^#^	mg/day	23 ± 2	23 ± 3	22 ± 2	NS		
Urea nitrogen excreted^#^	mg/day	374 ± 32	441 ± 33	335 ± 35	NS		
Creatinine nitrogen excreted^#^	*μ*g/day	54 ± 6^A^	67 ± 10^B^	79 + 5^B^	0.003		
NO_*x*_ nitrogen excreted^#^	*μ*g/day	2.7 ± 0.2^A^	3.3 ± 0.2^AB^	3.8 ± 0.4^B^	0.020		
Nitrogen excreted not accounted (N gap)^#^	mg/day	21 ± 25^A^	24 ± 14^A^	99 ± 15^B^	<0.001		

The values are the mean ± standard error of the mean (SEM) for 6 different animals. Statistical significance of the differences between groups: *P* (one-way analysis of variance (ANOVA): time); different superscript letters represent statistically significant differences between groups (Bonferroni *posthoc* test) *P < 0.05 for Student's *t*-test (30-day changes).^ #^Data between days 0 and 27.

**Table 2:** Organ and tissue weights of male rats fed a cafeteria diet for one month compared with controls fed a standard rat chow diet.

Tissue/organ	Control (g) (*n* = 6)	Cafeteria diet-fed (g) (*n* = 6)	*P*
Skeletal muscle	143 ± 4	164 ± 5	0.007
Skin	54.8 ± 1.14	55.5 ± 3.84	NS
Liver	9.86 ± 0.48	11.23 ± 0.38	NS
Small intestine	2.98 ± 0.22	2.90 ± 0.18	NS
Kidneys	2.14 ± 0.08	2.27 ± 0.06	NS
Brain	1.940 ± 0.073	1.884 ± 0.027	NS
Large intestine	1.46 ± 0.05	1.31 ± 0.06	NS
Lungs	1.418 ± 0.164	1.308 ± 0.041	NS
Stomach	1.265 ± 0.039	1.438 ± 0.054	0.023
Heart	0.936 ± 0.048	1.075 ± 0.024	0.027
Interscapular BAT	0.413 ± 0.042	0.442 ± 0.041	NS
Subcutaneous WAT	5.96 ± 0.69	9.11 ± 1.11	0.015
Mesenteric WAT	3.61 ± 0.52	3.86 ± 0.09	NS
Retroperitoneal WAT	2.58 ± 0.36	4.66 ± 0.36	0.002
Epididymal WAT	1.53 ± 0.26	4.43 ± 0.38	<0.001
Pericardial WAT	0.252 ± 0.047	0.311 ± 0.041	NS
Sum of five WAT sites	13.93 ± 1.89	22.23 ± 1.78	**0.007**

The values are the mean ± SEM for 6 different animals. BAT: brown adipose tissue; WAT: white adipose tissue. Statistical significance of the differences between groups; *P* was calculated with Student's *t* test.

**Table 3:** Plasma nitrate and nitrite concentrations of male rats fed a cafeteria diet for one month compared with controls fed a standard rat chow diet.

	Units	Control	Cafeteria diet-fed
Nitrite	*μ*M	3.4 ± 1.6	3.3 ± 1.3
Nitrate	*μ*M	48.0 ± 4.7	45.6 + 6.6
Nitrate	% of total	94.5 ± 2.6	92.8 ± 2.8
NO_*x*_ total	*μ*M	51.4 ± 6.0	44.8 ± 7.3

The values are the mean ± SEM for 6 different animals. There were no significant differences between the two groups (*P* > 0.05, Student's *t*-test) for any parameter. WAT: white adipose tissue, BAT: brown adipose tissue.

**Table 4:** Plasma amino acid concentrations of male rats fed a cafeteria diet for one month compared with controls fed a standard rat chow diet.

Amino acid	Control (*μ*M)	Cafeteria diet-fed (*μ*M)	*P*
Alanine	464 ± 5	455 ± 37	NS
Aspartate*	31 ± 3	40 ± 8	NS
Asparagine*	49 ± 5	57 ± 4	NS
Σ Asp + Asn	81 ± 4	87 ± 8	**NS**
Glutamate*	149 ± 17	160 ± 11	NS
Glutamine*	487 ± 27	582 ± 5	0.005
Σ Glu + Gln	641 ± 48	705 ± 32	**NS**
Proline	139 ± 12	152 ± 12	NS
Hydroxyproline	22 ± 3	25 ± 4	NS
Threonine	165 ± 10	202 ± 7	0.010
Serine	186 ± 8	245 ± 13	0.003
Glycine	342 ± 9	417 ± 17	0.002
Leucine*	144 ± 7	146 ± 10	NS
Isoleucine*	52 ± 4	61 ± 8	NS
Valine*	157 ± 14	162 ± 15	NS
Σ branched chain	355 ± 36	355 ± 15	**NS**
Phenylalanine	76 ± 4	87 ± 6	NS
Tyrosine	87 ± 3	93 ± 6	NS
Tryptophan	95 ± 11	101 ± 7	NS
Methionine	51 ± 3	53 ± 4	NS
Cysteine	21 ± 3	22 ± 2	NS
Lysine	376 ± 18	396 ± 14	NS
Histidine	62 ± 4	69 ± 8	NS
Ornithine*	50 ± 3	68 ± 7	0.045
Citrulline*	48 ± 4	51 ± 4	NS
Arginine*	209 ± 10	164 ± 8	0.006
Σ urea cycle	278 ± 12	305 ± 14	**NS**
Σ total (mM)	3.98 ± 0.10	4.22 ± 0.19	**NS**

The values are the mean ± SEM for 6 different animals. Statistical significance of the differences between the two groups was determined with Student's *t*-test. NS: *P* > 0.05. Asterisks “*” indicate the amino acids incorporated in the sums marked in bold below them.

**Table 5:** Plasma metabolite levels of male rats fed a cafeteria diet for one month compared with controls fed a standard rat chow diet.

Plasma values (mM)	Control	Cafeteria diet-fed	*P*
Glucose	8.13 ± 0.36	9.20 ± 0.28	0.042
Triacylglycerols	1.91 ± 0.06	1.84 ± 0.08	NS
Cholesterol	1.75 ± 0.17	1.90 ± 0.17	NS
Urea	5.28 ± 0.24	3.94 ± 0.21	0.002

The values are the mean ± SEM for 6 different animals. Statistical significance of the differences between both groups; *P* was determined with Student's *t-*test.
